# Multiomics analysis reveals the potential mechanism of high‐fat diet in dextran sulfate sodium‐induced colitis mice model

**DOI:** 10.1002/fsn3.4426

**Published:** 2024-08-30

**Authors:** Yuyang Zhao, Zhimin Chen, Ruiyi Dong, Yufan Liu, Yixin Zhang, Yan Guo, Meiyi Yu, Xiang Li, Jiangbin Wang

**Affiliations:** ^1^ Department of Gastroenterology China‐Japan Union Hospital of Jilin University Changchun Jilin China; ^2^ Department of Pharmacology College of Basic Medical Sciences, Jilin University Changchun Jilin China; ^3^ College of Physical Education, Hunan Normal University Changsha China

**Keywords:** DSS, gut microbiota, HFD, multiomics analysis, RNA‐seq

## Abstract

A high‐fat diet (HFD) is recognized as an important contributor to inflammatory bowel disease (IBD). However, the precise underlying mechanism of HFD on IBD remains elusive. This study aimed to investigate the potential mechanism by which HFD affects IBD using 16S rRNA‐sequencing and RNA‐seq technology. Results indicated that HFD‐treated mice exhibited notable alternations in the structure and composition of the gut microbiota, with some of these alternations being associated with the pathogenesis of IBD. Analysis of the colon transcriptome revealed 11 hub genes and 7 hub pathways among control, DSS‐induced colitis, and HFD + DSS‐treated groups. Further analysis explores the relationship between the hub pathways and genes, as well as the hub genes and gut microbiota. Overall, the findings indicate that the impact of HFD on DSS‐induced colitis may be linked to intestinal dysbiosis and specific genes such as *Abca8b*, *Ace2*, *Apoa1*, *Apoa4*, *Apoc3*, *Aspa*, *Dpp4*, *Maob*, *Slc34a2*, *Slc7a9*, and *Trpm6*. These results provide valuable insights for determining potential therapeutic targets for addressing HFD‐induced IBD.

## Graphical abstract

HFD‐treated mice exhibited notable alternations in the structure and composition of the gut microbiota, with some of these alternations being associated with the pathogenesis of IBD. Analysis of the colon transcriptome revealed 11 hub genes and 7 hub pathways among control, DSS‐induced colitis, and HFD + DSS‐treated groups. Impact of HFD on DSS‐induced colitis may be linked to intestinal dysbiosis and specific genes such as Abca8b, Ace2, Apoa1, Apoa4, Apoc3, Aspa, Dpp4, Maob, Slc34a2, Slc7a9, and Trpm6.

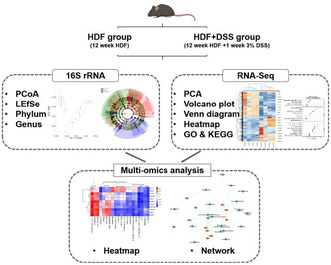

## 1 INTRODUCTION

Inflammatory bowel disease (IBD) is a collection of chronic inflammatory disorders impacting the gastrointestinal tract, such as Crohn's disease and ulcerative colitis (Torres et al., 2017). The incidence of IBD has been steadily increasing worldwide, making it a crucial global health concern (Xavier & Podolsky, 2007). The development of IBD is influenced by various factors, such as genetic susceptibility (McGovern et al., 2015), external environmental (Piovani et al., 2019), and microbial factors (Zuo & Ng, 2018). Most recent research has found that a high‐fat diet (HFD) increases the risk of IBD (Voutilainen et al., 2018). Some large‐scale prospective cohort studies showed that non‐alcoholic fatty liver disease (NAFLD) is associated with an increased risk of incident IBD (Wang et al., 2024; Zhang et al., 2023) and worse hospitalization outcomes in IBD patients (Chen et al., 2023; Hyun et al., 2024; Noorian et al., 2022). However, the underlying mechanism is still inadequately characterized.

The intestinal microbiota has been shown to play a remarkable role in the pathogenesis of various diseases (Ananthakrishnan et al., 2024). Imbalances in the gut microbiota, known as dysbiosis, have been linked to the development and progression of IBD. The microbiota is predominantly distributed in the colon and lives on the surface of the intestinal mucosa, forming a bacterial biofilm that impacts intestinal nutrient metabolism, permeability, and immune system function (Shen et al., 2018). Studies have reported that patients with IBD have a slightly diverse range of microorganisms in their gut compared to healthy individuals (Joossens et al., 2011). Furthermore, research has shown that the microbiota in IBD patients is less stable and more prone to fluctuations compared to healthy individuals (Andoh et al., 2011). Changes in the structure and function of the gut microbiota have been linked to metabolic disorders due to HFD (Agus et al., 2021). These disruptions in the microbiota contribute to the development of other diseases. The correlation between elevated intestinal permeability and NAFLD has been consistently supported by numerous clinical and experimental findings (Luther et al., 2015; Massier et al., 2021). Determining the effect of HFD on IBD may be mediated by the intestinal microbiota.

The gut microbiota plays a crucial role in preserving the balance and stability of the intestines, known as intestinal homeostasis, and regulating host gene expression. The diverse microbial community in the gut can interact with host cells and influence gene expression through various mechanisms, impacting various physiological processes and overall health. This intricate relationship highlights the importance of the gut microbiota in normal functioning and disease development (Lin & Zhang, 2017; Song & Chan, 2019). Recent studies have shown a strong correlation between *Escherichia_Shigella* and *Lepr*, a gene associated with liver injury and inflammatory response (Zhao et al., 2019). Transcriptomic technologies, such as RNA sequencing and microarray analysis, have revolutionized the field of genomics by allowing researchers to study the expression levels of thousands of genes simultaneously, offering valuable insights into the molecular mechanisms that underlie a range of biological processes and diseases. Researchers can identify potential drug targets, biomarkers for disease diagnosis, and pathways that can be targeted for therapeutic intervention by analyzing gene functions and signaling pathways. The use of transcriptomic technologies in clinical diagnosis and drug development has notably advanced our understanding of disease mechanisms and has the potential to lead to the development of highly targeted and effective treatments (Wang et al., 2009). Conducting transcriptome sequencing analysis on colitis induced by an HFD may help elucidate the underlying pathogenic mechanisms.

This study utilized an HFD‐induced NAFLD model and a dextran sulfate sodium (DSS)‐induced IBD model. Comparisons were conducted among groups exposed to an HFD, an HFD with DSS, and a healthy control group by leveraging 16S rRNA sequencing for microbiome analysis and RNA sequencing to examine differential gene expression in the colon. These comparisons aimed to uncover the signaling pathways and molecular mechanisms associated with HFD‐induced colon injury. Furthermore, the study explored the correlations between gut microbes and gene expressions in the colon to explore the potential link between bacteria taxa and host gene expression in the development of IBD following HFD pretreatment. These findings aim to advance our understanding of the impact of HFD on IBD and may reveal new therapeutic targets for HFD‐induced IBD treatment.

## 2 MATERIALS AND METHODS

### 2.1 Chemicals and reagents

DSS was procured from MP Biomedicals (Solon, OH, United States). Other reagents utilized were purchased from Sigma‐Aldrich (St. Louis, MO, USA). The Prime Script™ RT Master Mix was purchased from TaKaRa Biological Engineering Company (Da Lian, China). SYBR Green Master was obtained from Roche (Foster City, United States). The high‐fat diet (HFD, D12492), containing 60% of its energy from fat, and the control chow diet (CD, D12450B) were supplied by Beijing Hua Fu Kang Biotechnology Co. Ltd. (Beijing, China).

### 2.2 Animal models

All animal experiments were conducted in compliance with the Jilin University Animal Care and Use Committee and the Animal Experimentation Ethics Committee's approval (No. SY202302020). Six‐ to eight‐week‐old male C57BL/6J mice (weight 20 ± 2 g) were sourced from Beijing Hua Fu Kang Biotechnology Co. Ltd. (Beijing, China) and acclimated under controlled environmental conditions including room temperature (22 ± 2°C), humidity (40%–70%), and a 12‐h light/12‐h dark cycle for a minimum of 1 week prior to the experiments. Each mouse was weighed on Day 0 and then randomly assigned to one of three groups: control (Con.), DSS, and HFD + DSS, with eight mice in each group. The HFD + DSS group received an HFD for 12 weeks, while the control and DSS groups were fed a standard CD. Subsequently, the mice in the DSS and HFD + DSS groups received 3% DSS in their drinking water for 7 consecutive Days in 12th week while maintaining their respective diets. Mouse body weights were recorded every 2 weeks, and the animals were euthanized at week 12 after DSS treatment. Liver tissue, colon tissue, and fecal samples were collected for further analysis.

### 2.3 Immunohistochemistry analysis

After euthanasia, the colon and liver of the mice were promptly removed, and colon length was then recorded for each mouse. Subsequently, paraffin‐embedded colonic samples were sectioned and stained with hematoxylin and eosin (H&E). The stained sections were viewed and analyzed using a Leica DM3000 microscope from LEICA in Wetzlar, Germany. The severity of colitis was monitored by calculating disease activity index (DAI) scores by assessing body weight loss, stool consistency, and rectal bleeding, following a previously outlined methodology with some modifications (Guo et al., 2022). The scoring criteria are as follows: weight loss is scored as 0—0%, 1—1% to 5%, 2—5% to 10%, 3—10% to 20%, and 4—over 20%; diarrhea is rated as 0—normal, 2—loose stools, and 4—diarrhea; and rectal bleeding is classified as 0—normal and 4—severe bleeding.

### 2.4 16S rRNA sequencing and analysis

The colon samples were taken and immediately frozen in liquid nitrogen. The samples were then placed in storage at −80°C until they could undergo 16S rRNA sequencing with established protocols (Li et al., 2022). Genomic DNA was isolated from the gut content samples and utilized for PCR amplification of the bacterial 16S rDNA gene. The V3–V4 regions of the 16S rDNA (343F: TACGGRAGGCAGCAG, 798R: AGGGTATCTAATCCT) were specifically targeted for amplification. The resulting amplicons were visualized by electrophoresis which was performed on a 1% agarose gel and subsequently sequenced using the Illumina MiSeq System (Illumina, California, USA). The sequence data were clustered to generate operational taxonomic units (OTUs) at 97% similarity through the use of VSEARCH software. Representative reads from each OTU were chosen using the QIIME package and annotated by conducting a BLAST search against the Silva database Version 123 with the RDP classifier at a 70% confidence threshold. Principal coordinate analysis (PCoA) was conducted using the weighted UniFrac distance algorithm (Lozupone & Knight, 2005).

### 2.5 RNA sequencing and data analysis

The samples for the RNA‐sequencing analysis were processed using established methods that were implemented in previous studies (Li et al., 2024). First, RNA was extracted from four biological replicates of colon tissues using the TRIzol kit (Invitrogen, United States). The quality of the RNA samples was assessed using a NanoDrop 2000 spectrophotometer to measure the absorbance and concentrations. Subsequently, sequencing libraries were prepared using the MGIEasy Universal Library Convert Kit, the VAHTS® Universal V6 RNA‐seq Library Prep Kit for Illumina, and VAHTS® mRNA Capture Beads following the specific protocols provided by the respective manufacturers. Principal component analysis (PCA) was performed to assess the overall variance and relationships between different samples. Furthermore, differential gene expression analysis was conducted using the DESeq (2012) R package to compare the gene expression levels between different groups, enabling the identification of genes that are significantly upregulated or downregulated. In the differential gene expression analysis, genes with a fold change of ≥2 were considered to be significantly differentially expressed.

### 2.6 Enrichment analysis

Gene Ontology (GO) and Kyoto Encyclopedia of Genes and Genomes (KEGG) enrichment analyses are commonly used to provide functional insights into differentially expressed genes (DEGs). GO analysis helps categorize genes based on biological process, cellular component, and molecular function (Ashburner et al., 2000). The annotations for this analysis are typically sourced from GO (www.geneontology.org/) and NCBI (www.ncbi.nlm.nih.gov/). Meanwhile, KEGG pathway analysis identifies pathways enriched with DEGs, providing a broad perspective on the biological importance of gene expression changes, utilizing the KEGG database (www.genome.jp/kegg/) (Kanehisa et al., 2008). GO and KEGG enrichment analyses in R leverage the hypergeometric distribution to assess the statistical significance of the overlap between DEGs and specific GO terms or pathways. This method helps in determining whether the observed overlap is more significant than what would be expected by random chance, providing valuable insights into the biological relevance of gene expression changes.

### 2.7 qRT‐PCR validation

In the qRT‐PCR analysis, RNA was isolated using the TRIzol reagent protocol. Subsequently, first‐strand cDNA synthesis was conducted using the Prime Script™ RT Master Mix. Real‐time PCR was performed in a 20 μL reaction volume (2 μL cDNA, 10 μL SYBR Green, 1 μL of forward and reverse primers (10 μM), and RNase‐free water) using the QuantStudio Real‐Time PCR System. The mRNA expression levels were then analyzed using the 2^−ΔΔCT^ method, with the target mRNA levels normalized to the GAPDH mRNA expression. Primers for the 11 hub genes were designed using Primer‐BLAST as shown in Table S1.

### 2.8 Statistical analysis

All experiments were independently replicated a minimum of three times to validate the results and ensure consistency and reliability. Data analyses were conducted using GraphPad Prism software (version 8.0). The data were summarized as mean ± standard deviation and compared using Student's *t*‐test for two‐group comparisons and one‐way ANVOA with a Tukey test for multiple comparisons. Statistical significance was determined for all tests with *p* < .05.

## 3 RESULTS

### 3.1 HFD aggravates DSS‐induced colitis in mice

**FIGURE 1:**
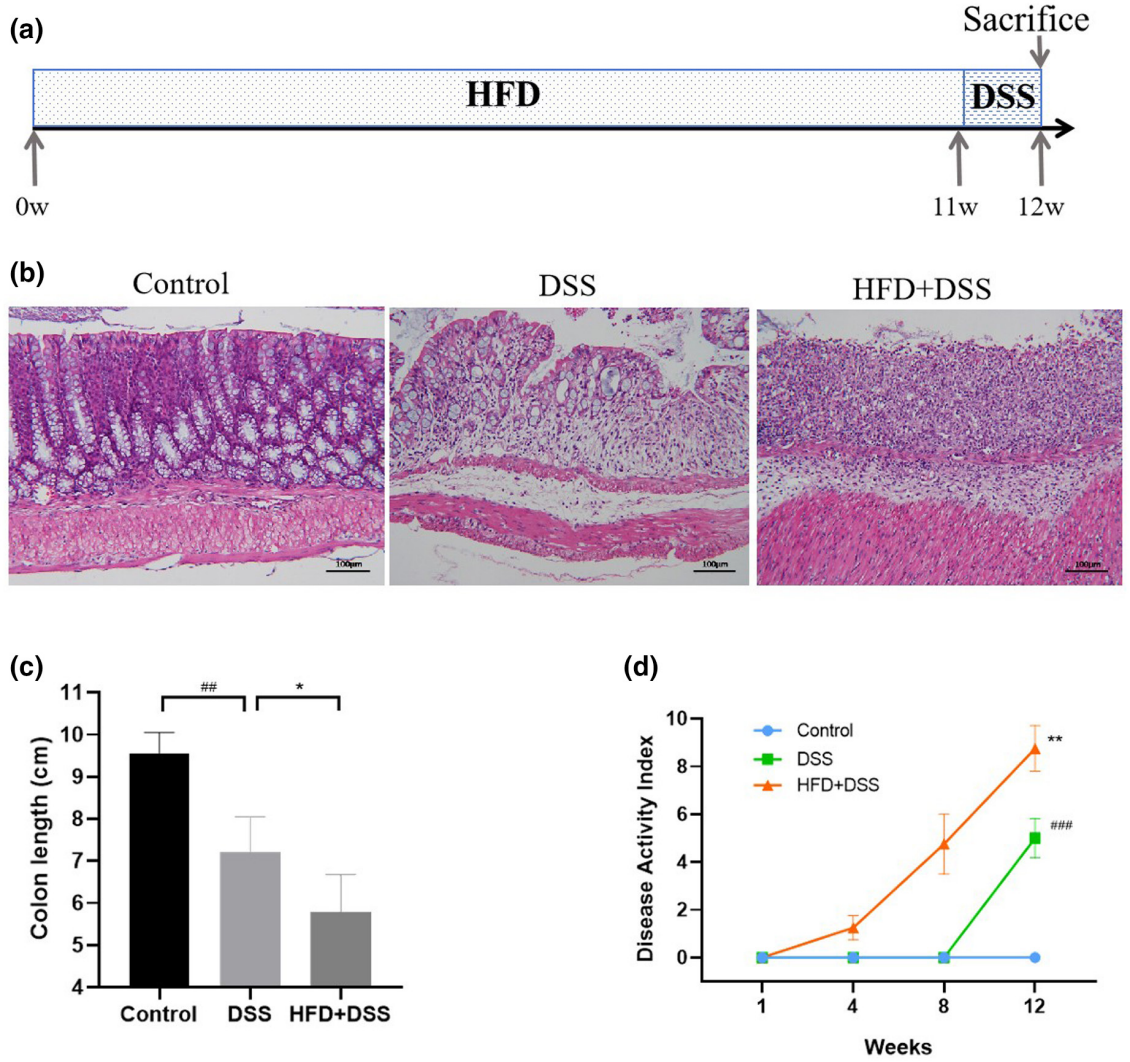
Effect of an HFD on DSS‐induced colitis in mice. (a) Diagram of the animal model; (b) H&E‐staining images of colon; (c) bar graphs of colon length; (d) disease activity index of mice. (*n* = 5, scale bar = 100 μm, compared to control, *p < .05, **p < .01; compared to DSS, ^##^ *p* < .01, ^###^ *p* < .001.)

Mice were fed an HDF for 12 weeks and provided with sterilized water containing 3% DSS in the last week to examine the effect of this diet on the DSS‐induced colitis mouse model (Figure 1a). The pathologic changes were determined using H&E staining (Figure 1b). In comparison to the DSS group, the HFD + DSS group exhibited pronounced epithelial mucosal loss, glandular atrophy, infiltration of inflammatory cells, and severe crypt injury. As shown in Figure 1c,d, mice treated with DSS experienced a notable shortening of colonic length and a marked increase in DAI when compared to the control group. The colon lengths were shortened and the DAI was significantly increased in the HFD + DSS group compared with those in the DSS group. Overall, the findings above indicate that an HFD triggered a notable inflammatory reaction in the DSS‐induced mice.

### 3.2 Dysbiosis of gut microbiota caused by HFD

**FIGURE 2:**
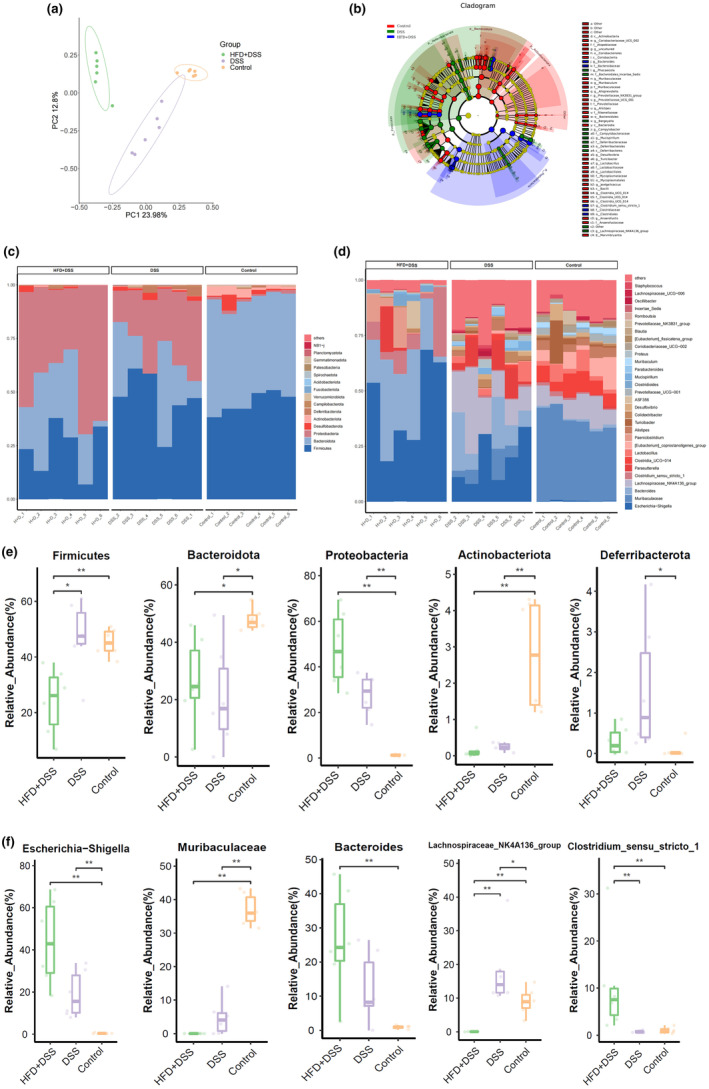
HFD alters the composition of the gut microbiota. (a) PCoA; (b) LEfSe analysis; (c) structure and composition of the gut microbiota at the phylum level; (d) structure and composition of the gut microbiota at the genus level; (e) relative abundance of different microbial taxa at the phylum; (f) relative abundance of different microbial taxa at the genus levels. (*n* = 6, *p < .05, **p < .01).

Dysbiosis of gut bacteria is recognized as one of the contributing factors to the development of colitis: this imbalance in the gut microbiota can disrupt the delicate ecosystem of bacteria in the gut, leading to inflammation and other gastrointestinal issues (Alshehri et al., 2021). Therefore, this study further explored the alternations in gut microbiota in DSS‐induced mice with HFD regimens. The results of PCoA (Figure 2a) revealed distinct differences in bacterial community structure among the control, DSS, and HFD + DSS groups, indicating unique compositions and diversity profiles among the groups and highlighting the diverse nature of microbiota populations. The subsequent LEfSe analysis cladogram plots (Figure 2b) indicated remarkable differences in microbiota structures among the three groups. Further analysis at the phylum and genus levels revealed that *Firmicutes* and *Bacteroidota* were the most abundant phyla in all groups (Figure 2c,d). The top five bacteria with the most notable changes at the phylum and genus levels were depicted in Figure 2e,f. At the phylum level (Figure 2e), the relative abundance of *Firmicutes* was substantially lower in the HFD + DSS group compared to the DSS group. Additionally, the relative abundance of Bacteroidota and Actinobacteriota was both lower in the DSS and HFD + DSS groups compared to the control group. At the genus level (Figure 2f), the relative abundance of *Lachnospiraceae_NK4A136_group* was higher in the DSS group but notably lower in the HFD + DSS group compared to the control group. These findings indicate that HFD can have a profound effect on the structure and composition of the gut microbiota in mice with DSS‐induced colitis, potentially playing a role in the development or exacerbation of intestinal inflammation.

### 3.3 HDF modulates the gene expression of colon in DSS‐induced mice

**FIGURE 3:**
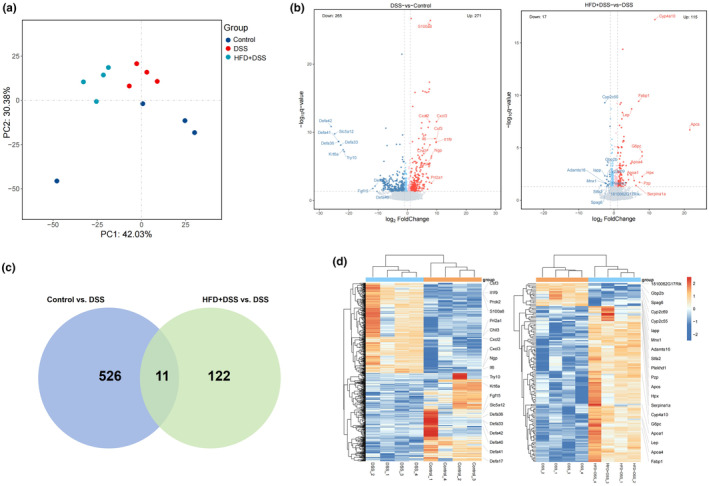
HFD induces changes in gene expression in mice with colitis. (a) PCA of the transcriptional profiling; (b) volcano plot (*p* < .05); (c) heatmap of GEGs in the colon among control, DSS, and HFD + DSS groups; (d) Venn diagram of control, DSS, and HFD + DSS groups. (*n* = 4).

RNA sequencing of colonic tissues was conducted to explore the impact of HFD on mouse colitis and comprehensively examine the role of colonic bacterial taxa in the specific biological processes and molecular mechanisms involved in the development of colitis. PCA revealed a notable difference among the gene expression profiles among the three groups (Figure 3a). The volcano plot illustrated the differences in gene expression levels between the control and DSS groups, as well as between the DSS‐treated and HFD + DSS groups (Figure 3b). Compared with the control group, 271 and 265 genes were upregulated and downregulated in the DSS group, respectively. Compared with the DSS group, 115 and 17 genes were upregulated and downregulated, respectively, in the HFD + DSS group. The Venn diagram showed that 11 hub genes were identified, which are the DEGs among three groups (Figure 3c, Table 1). The heatmap showed the expression variation of 11 hub genes (Figure 3d).

**TABLE 1:** Hub genes among control, DSS, and HFD + DSS groups.

	DSS‐versus‐con.	HFD + DSS‐versus‐DSS	
Gene_ID	Fold Change	Fold Change	Description
*Abca8b*	0.642	1.935	ATP‐binding cassette, sub‐family A (ABC1), member 8b
*Ace2*	0.025	4.933	Angiotensin I‐converting enzyme (peptidyl‐dipeptidase A) 2
*Apoa1*	0.003	53.469	Apolipoprotein A‐I
*Apoa4*	0.004	260.497	Apolipoprotein A‐IV
*Apoc3*	0.003	28.122	Apolipoprotein C‐III
*Aspa*	0.058	2.265	Aspartoacylase
*Dpp4*	0.089	2.749	Dipeptidylpeptidase 4
*Maob*	0.370	1.852	Monoamine oxidase B
*Slc34a2*	0.057	5.000	Solute carrier family 34 (sodium phosphate), member 2
*Slc7a9*	0.055	3.543	Solute carrier family 7 (cationic amino acid transporter, y + system), member 9
*Trpm6*	0.166	3.139	Transient receptor potential cation channel, subfamily M, member 6

### 3.4 PCR analysis of hub genes

**FIGURE 4:**
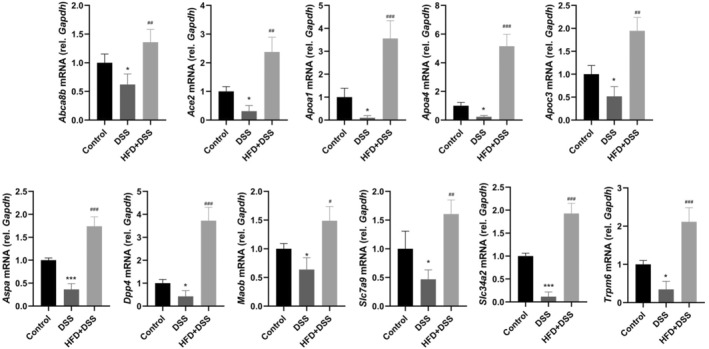
Expression of the hub genes was validated by qRT‐PCR. (*n* = 4, compared to control, *p < .05, ***p < .001; compared to DSS, ^#^ *p* < .05, ^##^ *p* < .01, ^###^ *p* < .001).

The validity of the RNA‐seq results was further confirmed through qRT‐PCR analysis, which aimed to confirm the expression patterns of hub genes identified in the RNA‐seq and DEG analyses. The current research revealed that the mRNA expressions of 11 hub genes, including *Abca8b*, *Ace2*, *Apoa1*, *Apoa4*, *Apoc3*, *Aspa*, *Dpp4*, *Maob*, *Slc34a2*, *Slc7a9*, and *Trpm6*, were significantly lower compared to the control group (Figure 4). Moreover, with HFD pretreatment, the mRNA expression levels of these hub genes were notably lower in the HFD + DSS group compared to the DSS group. Importantly, the relative mRNA expression levels of the 11 hub genes confirmed by qRT‐PCR were consistent with those obtained from RNA‐seq. This consistency indicates the reliability and reproducibility of the RNA‐seq findings.

### 3.5 GO enrichment analysis

**FIGURE 5:**
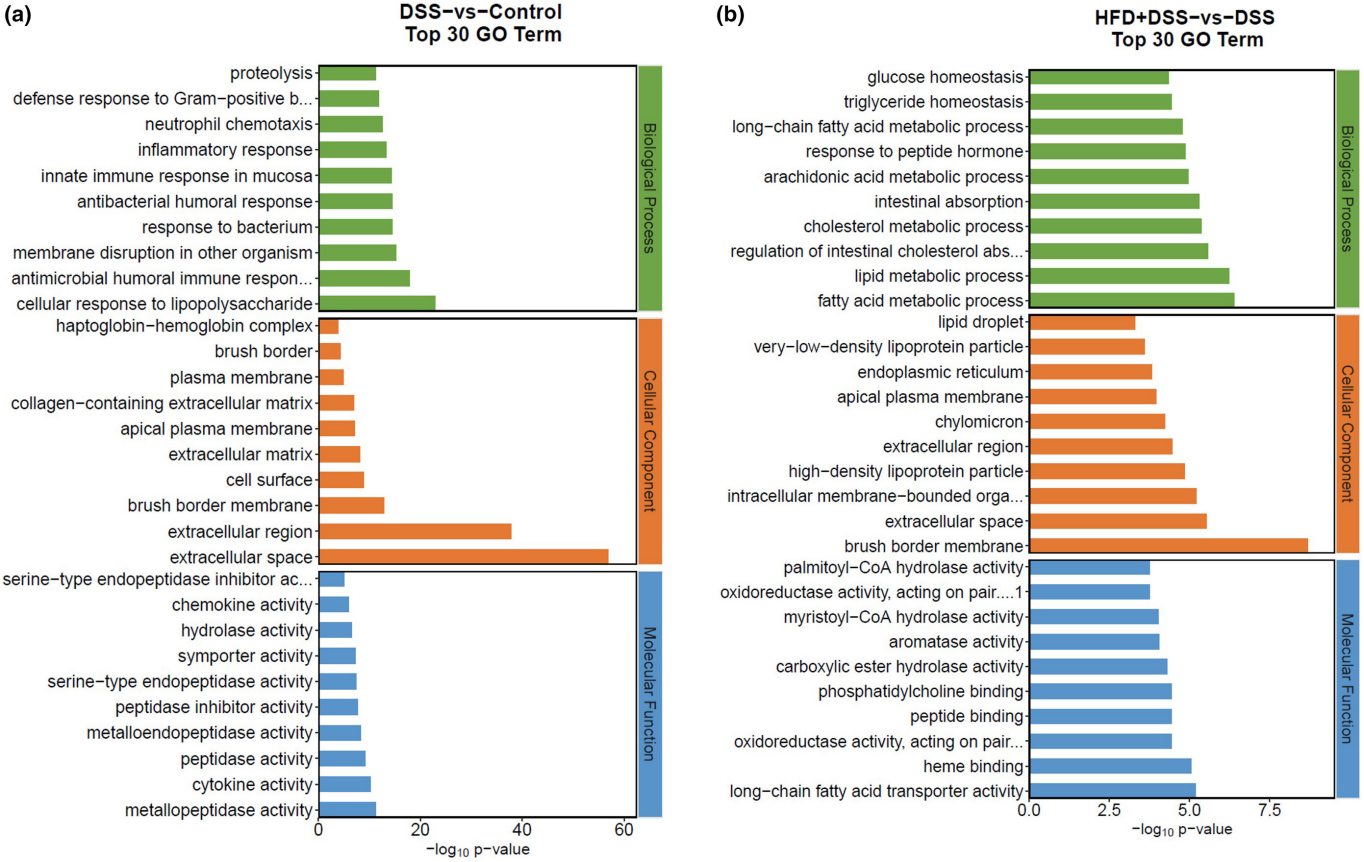
Gene ontology enrichment analysis. (a) GO analysis between the control and DSS groups; (b) GO analysis between the DSS and HFD + DSS groups.

Conducting a GO enrichment analysis can provide insights into pathways that are closely associated with the progression of IBD. As shown in Figure 5a, compared to the control and DSS groups, the notably different pathways were primarily related to cellular response to lipopolysaccharide, extracellular space, and metallopeptidase activity in terms of biological process, cellular component, and molecular function, respectively. When compared between HFD + DSS and DSS groups, the top three GO terms of most genes included fatty acid metabolic process, brush border membrane, and long‐chain fatty acid transporter activity (Figure 5b).

### 3.6 KEGG functional enrichment analysis

**FIGURE 6:**
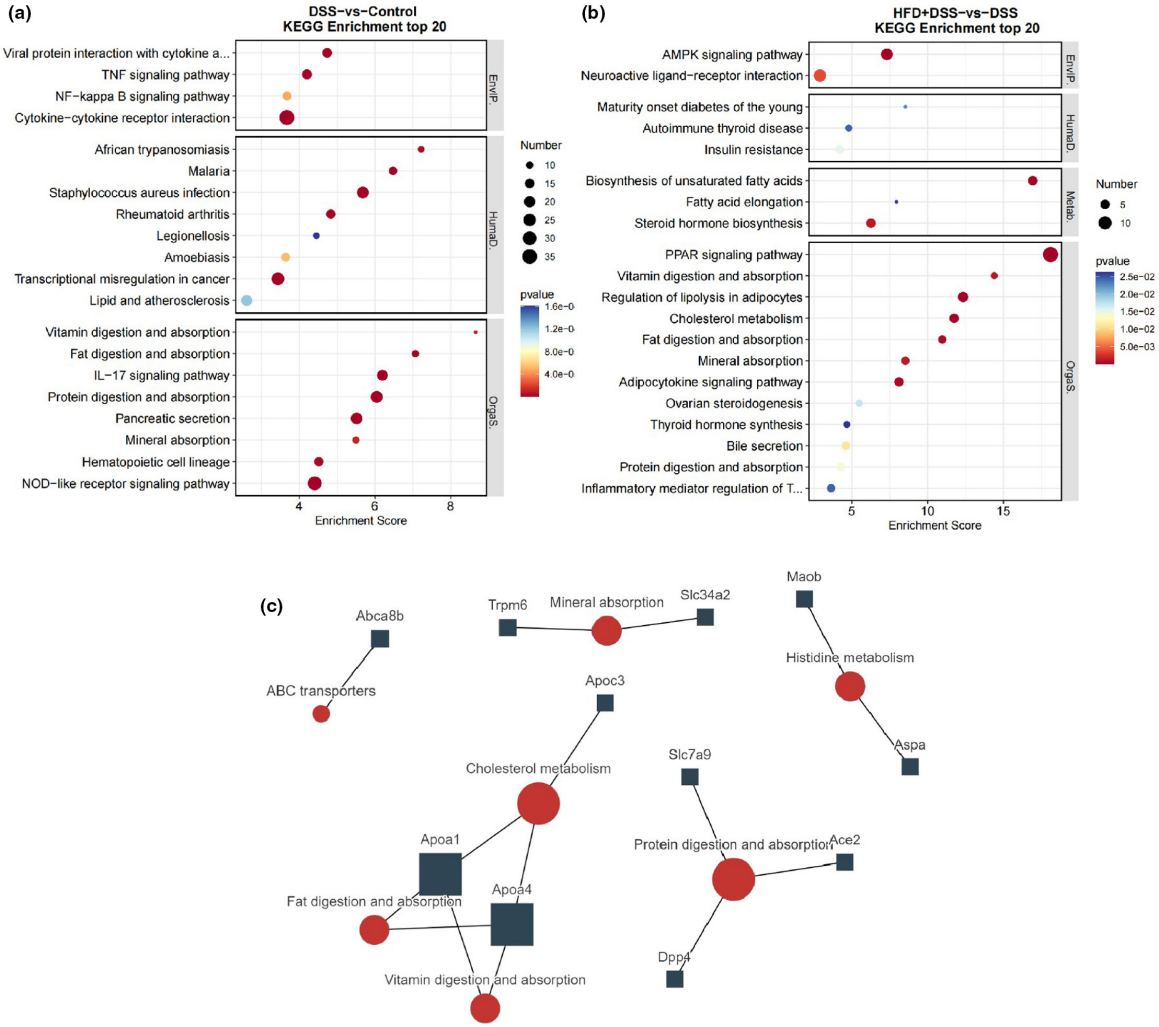
KEGG signaling pathway enrichment analysis results. (a) KEGG analysis between the control and DSS groups; (b) KEGG analysis between the DSS and HFD + DSS groups; (c) correlations between hub pathways and genes among the control, DSS, and DSS + HFD groups.

KEGG analysis is an effective way to determine the key pathways involved in the biological process. As shown in Figure 6a,b, the top three signaling pathways between control and DSS groups are cytokine–cytokine receptor interaction, NOD‐like receptor signaling pathway, and transcriptional misregulation in cancer; the top three signaling pathways between HFD + DSS and DSS groups are PPAR signaling pathway, neuroactive ligand–receptor interaction, and AMPK signaling pathway. After comprehensive analysis across the three groups, seven hub pathways were identified, as shown in Table 2. In addition, the relationship between hub genes and pathways was analyzed as shown in Figure 6c.

**TABLE 2:** Hub pathways and involved hub genes of control, DSS, and HFD + DSS groups.

Term	Hub genes
Histidine metabolism	*Aspa*;*Maob*
ABC transporters	*Abca8b*
Protein digestion and absorption	*Ace2*;*Dpp4*;*Slc7a9*
Fat digestion and absorption	*Apoa1*;*Apoa4*
Vitamin digestion and absorption	*Apoa1*;*Apoa4*
Mineral absorption	*Slc34a2*;*Trpm6*
Cholesterol metabolism	*Apoa1*;*Apoa4*;*Apoc3*

### 3.7 Correlation between imbalanced gut microbiota and DEGs in the colon

**FIGURE 7:**
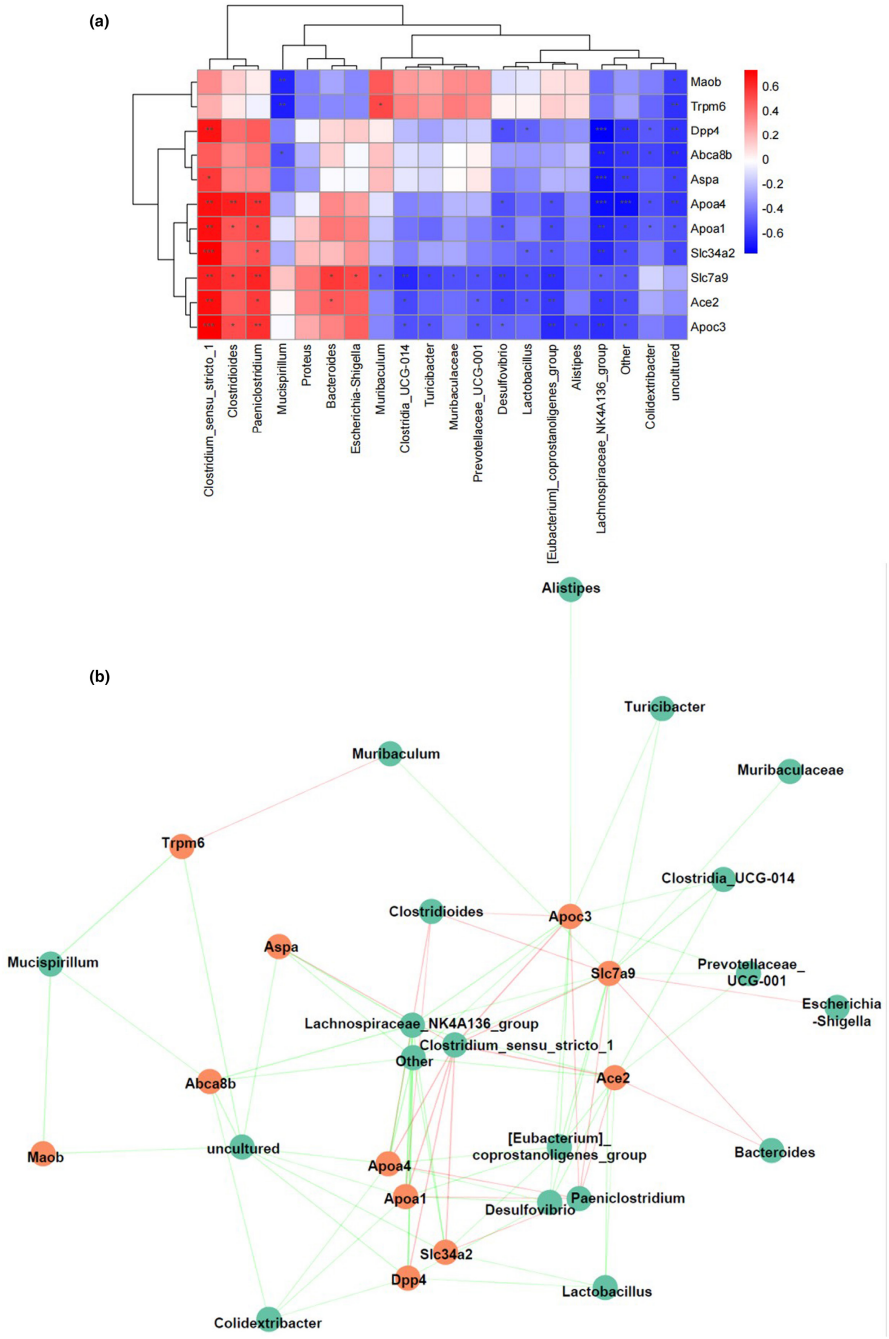
Gene–microbe correlations. (a) Heatmap; (b) network.

A multiomics analysis was performed to investigate the relationship between gut microbiota and genes altered by HFD. The heatmap and the network (Figure 7a,b) show the relationship between 11 hub genes and gut microbiota at the genus level.

## 4 DISCUSSION

HFD mouse models are widely used in the research of NAFLD because they mimic the steatosis, inflammation, and hepatic fibrosis typically found in this complex human disease (Nevzorova et al., 2020). In this study, based on previous reports, mice were fed with an HFD for 12 weeks to stimulate the condition of NAFLD (Li et al., 2020). DSS is a commonly used method due to its simplicity, reproducibility in inducing colonic lesions, and capability to mimic the clinical and histological features of IBD (Elson et al., 1995). Mice were provided with sterilized water containing 3% DSS for 1 week following the HFD regimen to assess its impact on IBD. The results showed that HFD aggravated visible colitis phenotypes, as evidenced by increased DAI, shortened colon length, and exacerbated pathological changes. 16S rRNA sequencing and RNA‐seq of colon tissue were performed to further explore the underlying mechanism.

HFD is crucial in maintaining gut homeostasis, and emerging evidence indicates the influence of the gut microbiota on IBD. However, the relationship between the altered gut microbiota due to HFD and IBD remains unknown. In the healthy intestine, the *Firmicutes* bacteria are dominant and help produce essential metabolic substances for the intestinal epithelium. However, in IBD, a noticeable decrease is observed in the abundance of *Firmicutes* in the microbiota (Martinez et al., 2008). This study revealed that HFD notably changed the structure and composition of the microbiota. Particularly, the relative abundance of *Firmicutes* was substantially lower in the HFD + DSS group compared with that in the DSS group, indicating that the effect of HFD may be mediated by *Firmicutes*. Understanding the role of the gut microbiota in mediating the effects of an HFD can provide valuable insights into the mechanisms underlying diet‐induced metabolic disturbances and help develop targeted interventions to restore microbial balance and improve metabolic health.

As previously reported, HFD can influence gene expression in the colon, and the intestinal microbiome plays a crucial role in interacting with host gene expressions (Dayama et al., 2020). Abnormal gene expressions are often closely associated with the development of various diseases (Yu et al., 2019). Therefore, in this study, RNA‐seq of the colon was conducted to analyze the gut microbe–host gene interactions. This approach allows for a comprehensive understanding of the influence of the intestinal microbiome on host gene expression in the context of the HFD‐induced exacerbation of IBD.

Transcriptomic studies showed that HDF notably increased the expression of the *Abca8b*, *Ace2*, *Apoa1*, *Apoa4*, *Apoc3*, *Aspa*, *Dpp4*, *Maob*, *Slc34a2*, *Slc7a9*, and *Trpm6* genes in colon tissue. The 11 hub genes were distributed in the 7 hub pathways, which included histidine metabolism, ABC transporters, vitamin digestion and absorption, protein digestion and absorption, mineral absorption, fat digestion and absorption, and cholesterol metabolism, increasing their relevance to the study of colitis.

Abca8b is part of a family of transporters that regulate lipid metabolism and cholesterol homeostasis (Trigueros‐Motos et al., 2017). Changes in the expression of Abca8b could affect the integrity of the intestinal barrier, contributing to the inflammation observed in colitis. Prior research has shown that a deficiency in ACE2 can lead to decreased absorption of certain dietary amino acids in the intestine (Penninger et al., 2021). Imbalances in ACE2 levels have been linked to chronic intestinal inflammation and diarrhea in mammals (Edwinson et al., 2021). Clinical research revealed that IBD patients have a higher ACE2:ACE ratio (Garg et al., 2020). Treatment with ACE inhibitors, such as captopril, has been associated with reduced surgery and hospitalization rates in individuals with IBD (Garg et al., 2020). In this study, HFD notably increased the RNA expression of ACE2, indicating HDF may aggravate IBD by affecting colon Ace2 expression. This hypothesis requires further exploration.

Additionally, the analysis showed that Apoa1 was markedly changed in the colon between HFD + DSS and DSS groups, and was involved in the identification of their hub pathways in this research. Apoa1 is not only an important protein constituent of high‐density lipoprotein but also plays a crucial role in modulating programmed cell death and inflammation (Kumar et al., 2020). Further research confirmed the involvement of Apoa1 in the JAK2/STAT3 and TLR5/MyD88/NF‐κB pathways (Chithra et al., 2017; Yiu et al., 2020). The exact mechanism of how HFD influences Apoa1 will be an interesting study. Research has shown that the levels of Apoa4 dramatically increase in response to the influx of fatty acids and triglycerides in the intestinal lumen (Liu et al., 2024). This increase is crucial for lipid transport and maintaining homeostasis. Recent studies indicate that the gut microbiota can modulate the expression of genes associated with lipid metabolism, including Apoa4 (Zhong et al., 2023). Changes in Apoa4 expression can be associated with dyslipidemia and increased cardiovascular risk.

The Aspa gene is involved in asparagine metabolism, which can influence cellular responses to stress and inflammation (Li & Lu, 2018). Aspa may also play a role in the immune response and cellular metabolism in the gut, thus contributing to or modulating the inflammatory processes observed in colitis. Ulcerative colitis (UC) is classified as a form of IBD (Danese & Fiocchi, 2011). DPP4 inhibitor sitagliptin has been shown to alleviate UC by affecting the GLP‐2 pathway (Ning et al., 2020). Additionally, a range of peptide DPP4 inhibitors have been confirmed to reduce colitis in mice through the same pathway (Salaga et al., 2017, 2018), indicating that the overexpression of intestinal DPP4 is closely related to IBD. This study showed that HFD increased the expression of colon DPP4. However, the effect of the DPP4 inhibitor on reducing the severity of colitis in HFD mice must be further confirmed. MAOB is an enzyme that breaks down neurotransmitters and is involved in metabolic pathways (Brosinsky et al., 2024). While primarily studied in neurobiology, MAOB may play a role in inflammation and oxidative stress in the gut (Tian et al., 2023), influencing the pathophysiology of colitis. Slc34a2 codes for a sodium‐phosphate cotransporter involved in phosphate transport (Ma et al., 2014). Phosphate handling is important for bone and gut health (Shobeiri et al., 2014), and dysregulation may impact gut inflammation and mineral homeostasis during colitis. TRPM6 is involved in Mg2+ homeostasis and has been linked to various cellular processes (Zou et al., 2020). Magnesium plays a role in inflammatory responses (Badaeva et al., 2024), and alterations in TRPM6 may affect magnesium levels in the gut, influencing inflammatory processes in colitis.

In addition to the analysis of RNA‐seq results, some connections between the microorganisms in the intestines and the DEGs in the colon were also observed. The relationship between gut microbiota and gene expression is a complex, bidirectional, and dynamic interplay that can considerably influence various aspects of human health and disease (Knights et al., 2014).

The gut microbiota produces various metabolites, such as short‐chain fatty acids (SCFAs), which are generated from the fermentation of dietary fibers (Jensen et al., 2024). SCFAs, such as butyrate, play a crucial role in regulating gene expression in colonocytes (Shen et al., 2023) They can influence pathways related to inflammation, cell proliferation, and apoptosis. In addition, gut bacteria can regulate gene expression through epigenetic mechanisms. Epigenetic modifications, such as DNA methylation and histone modifications, can influence gene expression patterns without altering the underlying DNA sequence (Allis & Jenuwein, 2016). Some studies have shown that the gut microbiota can influence host epigenetic modifications, leading to changes in gene expression profiles (Yano et al., 2015). Moreover, interactions between the gut microbiota and the host immune system can impact gene expression. Dysbiosis, or an imbalance in gut microbial populations, can trigger inflammatory responses that can alter gene expression in immune cells and other tissues, leading to chronic inflammation and autoimmune diseases (Manfredo Vieira et al., 2018). Specific gene polymorphisms related to immune response, metabolism, and mucosal barrier function may shape the microbial communities in the gut (Knights et al., 2014). Genes that encode for mucins (e.g., MUC2) and antimicrobial peptides can determine the gut environment and the types of bacteria that can colonize the colon (Pothuraju et al., 2021). A well‐functioning mucosal layer helps establish a balanced microbiota. Gene expression profiles associated with inflammatory responses can lead to shifts in microbiota composition. For example, an upregulation of inflammatory cytokines may create an environment conducive to pathogenic bacteria, while anti‐inflammatory profiles may favor beneficial microbes (Essex et al., 2024). In this research, nine hub genes were related to the *Lachnospiraceae_NK4A136_group*, eight hub genes were related to *Clostridium_sensu_stricto_1*, and six hub genes were related to *Desulfovibrio*. A total of 15, 11, and 10 categories of microbiota at the genus level were related to *Slc7a9*, *Apoc3*, and *Ace2* expressions, respectively.

SLC7A9 is a protein involved in the transport of amino acids across cell membranes (Fotiadis et al., 2013). Research on the specific relationship between SLC7A9 and the gut microbiota is limited. However, some studies showed that SLC7A9 and gut microbiota may involve the metabolism of certain amino acids by gut bacteria (Mardinoglu et al., 2015). The gut microbiota plays a crucial role in the breakdown and fermentation of dietary proteins, leading to the production of various metabolites, including amino acids (David et al., 2014). Thus, alterations in the composition of the gut microbiota may impact the availability of specific amino acids for transport by proteins such as SLC7A9. Disruptions in amino acid transport and metabolism could have implications for overall gut health and immune function. Therefore, further research is necessary to elucidate the specific interaction mechanisms between SLC7A9 and the gut microbiota and identify their potential implications for health and disease. Studying the interplay between amino acid transporters and the gut microbiota could provide valuable insights into the complex relationships among nutrient metabolism, host–microbiota interactions, and overall gut health.

ACE2 is a protein found on the surface of several different types of cells in the human body, including cells in the gut (Hashimoto et al., 2012). Some studies have found that the composition of the gut microbiota may influence the expression of ACE2 in the gut (Yeoh et al., 2021). SCFAs have been found to modulate ACE2 expression in the gut and SCFAs can upregulate ACE2 expression in the gut epithelium (Budden et al., 2017).

APOC3 plays a critical role in lipid metabolism by inhibiting lipoprotein and hepatic lipase, which are enzymes responsible for the breakdown of triglyceride‐rich lipoproteins (Dewey et al., 2017). Emerging evidence indicates potential interactions between APOC3 and the gut microbiota. APOC3 and gut microbiota involve the modulation of lipid metabolism and inflammation. Dysregulation of lipid metabolism, which is characterized by elevated levels of triglycerides and APOC3, is associated with metabolic disorders such as obesity, insulin resistance, and cardiovascular disease (Ginsberg et al., 2005). Recent studies have shown that the composition of the gut microbiota can influence lipid metabolism and potentially impact APOC3 levels (Caesar et al., 2016). Certain gut bacteria have been implicated in the production of short‐chain fatty acids (SCFAs) and bile acids, which can modulate lipid metabolism and inflammation in the host, respectively (den Besten et al., 2013). In addition, gut dysbiosis, which is characterized by an imbalance in microbial populations, has been associated with increased levels of circulating lipopolysaccharides (LPS) from gram‐negative bacteria. LPS can trigger inflammation and metabolic dysfunction, leading to altered APOC3 expression and lipid metabolism (Deopurkar et al., 2010). Further research is necessary to elucidate the specific mechanisms underlying the interplay between APOC3 and the gut microbiota and their impact on metabolic health. Understanding the relationship between APOC3 and the gut microbiota may offer new insights into the pathogenesis of metabolic disorders and potential therapeutic targets for modulating lipid metabolism and inflammation.

## 5 CONCLUSION

The findings revealed the effect of HDF in mouse models with DSS‐induced colitis. 16S rRNA sequencing revealed that HFD led to modification in the structure and composition of the gut microbiota in mice with DSS‐induced colitis, especially at phylum and genus levels. With pretreatment of HFD, the relative abundance of *Firmicutes* and *Lachnospiraceae_NK4A136_group* were both substantially lower in the HFD + DSS group compared to the DSS group. The relative abundance of *Clostridium_sensu_stricto_1* was significantly higher in the HFD + DSS group compared to the DSS group. Eleven hub genes, including *Abca8b*, *Ace2*, *Apoa1*, *Apoa4*, *Apoc3*, *Aspa*, *Dpp4*, *Maob*, *Slc34a2*, *Slc7a9* and *Trpm6*, were identified through transcriptomic analysis. These hub genes were significantly lower expressed in the DSS group but higher expressed in the HFD + DSS group. In addition, seven hub signaling pathways, including histidine metabolism, ABC transporters, protein digestion and absorption, fat digestion and absorption, vitamin digestion and absorption, mineral absorption, and cholesterol metabolism, were also identified among the control, DSS, and HFD + DSS groups. Overall, the findings establish a link between the gut microbiota and genes which may be the underlying mechanisms of exaggerated IBD pathology due to HFD.

## AUTHOR CONTRIBUTIONS

**Yuyang Zhao:** Data curation (equal); formal analysis (lead); investigation (equal); methodology (equal); writing – original draft (lead). **Zhimin Chen:** Formal analysis (equal); investigation (equal); methodology (equal); software (supporting). **Ruiyi Dong:** Formal analysis (equal); software (lead). **Yufan Liu:** Data curation (equal); investigation (equal); methodology (equal). **Yixin Zhang:** Data curation (equal); formal analysis (supporting); investigation (equal); methodology (supporting). **Yan Guo:** Formal analysis (equal); methodology (equal). **Meiyi Yu:** Methodology (equal). **Xiang Li:** Conceptualization (equal); funding acquisition (lead); supervision (equal); writing – review and editing (equal). **Jiangbin Wang:** Conceptualization (equal); resources (lead); supervision (equal); writing – review and editing (equal).

## FUNDING INFORMATION

This work was supported by Science and Technology Department of Jilin Province (grant number: 20240602079RC); Bethune Project of Jilin University (grant number: 2024B16); Li Xin Outstanding Young Teacher Training Program of Jilin University.

## CONFLICT OF INTEREST STATEMENT

The authors declare that they do not have any conflict of interest.

## Supporting information


**Table S1.** qRT‐PCR primer sequence of hub genes.

## Data Availability

The data that support the findings of this study are available on request from the corresponding author. The data are not publicly available due to privacy or ethical restrictions.
