# Supplementary figures and images for: Multiomics analysis reveals the potential mechanism of high‐fat diet in dextran sulfate sodium‐induced colitis mice model
Source: Food Sci Nutr. 2024 Aug 30;12(10):8309–23. doi: 10.1002/fsn3.4426 (PMC11521715; doi:10.1002/fsn3.4426)

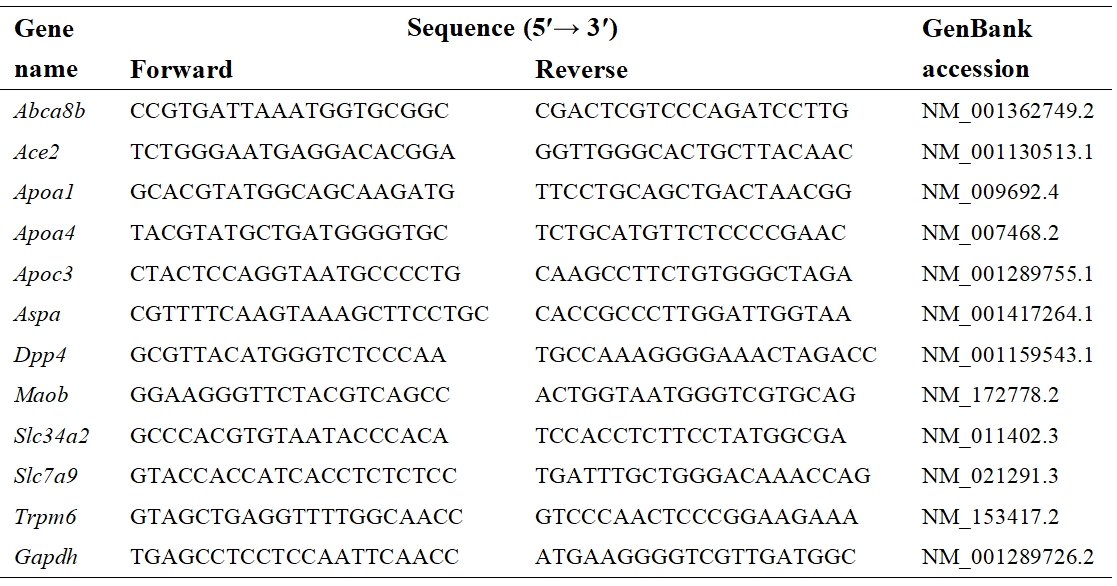

Supplement: Supplementary file 1 — Table S1. qRT‐PCR primer sequence of hub genes. [file FSN3-12-8309-s001.jpg]
